# Exploring Older Adults’ Beliefs About the Use of Intelligent Assistants for Consumer Health Information Management: A Participatory Design Study

**DOI:** 10.2196/15381

**Published:** 2019-12-11

**Authors:** Aqueasha Martin-Hammond, Sravani Vemireddy, Kartik Rao

**Affiliations:** 1 Department of Human-Centered Computing School of Informatics and Computing Indiana University-Purdue University Indianapolis Indianapolis, IN United States; 2 Department of Health Informatics School of Informatics and Computing Indiana University-Purdue University Indianapolis Indianapolis, IN United States

**Keywords:** intelligent assistants, artificial intelligence, chatbots, conversational agents, digital health, elderly, aging in place, participatory design, co-design, health information seeking

## Abstract

**Background:**

Intelligent assistants (IAs), also known as intelligent agents, use artificial intelligence to help users achieve a goal or complete a task. IAs represent a potential solution for providing older adults with individualized assistance at home, for example, to reduce social isolation, serve as memory aids, or help with disease management. However, to design IAs for health that are beneficial and accepted by older adults, it is important to understand their beliefs about IAs, how they would like to interact with IAs for consumer health, and how they desire to integrate IAs into their homes.

**Objective:**

We explore older adults’ mental models and beliefs about IAs, the tasks they want IAs to support, and how they would like to interact with IAs for consumer health. For the purpose of this study, we focus on IAs in the context of consumer health information management and search.

**Methods:**

We present findings from an exploratory, qualitative study that investigated older adults’ perspectives of IAs that aid with consumer health information search and management tasks. Eighteen older adults participated in a multiphase, participatory design workshop in which we engaged them in discussion, brainstorming, and design activities that helped us identify their current challenges managing and finding health information at home. We also explored their beliefs and ideas for an IA to assist them with consumer health tasks. We used participatory design activities to identify areas in which they felt IAs might be useful, but also to uncover the reasoning behind the ideas they presented. Discussions were audio-recorded and later transcribed. We compiled design artifacts collected during the study to supplement researcher transcripts and notes. Thematic analysis was used to analyze data.

**Results:**

We found that participants saw IAs as potentially useful for providing recommendations, facilitating collaboration between themselves and other caregivers, and for alerts of serious illness. However, they also desired familiar and natural interactions with IAs (eg, using voice) that could, if need be, provide fluid and unconstrained interactions, reason about their symptoms, and provide information or advice. Other participants discussed the need for flexible IAs that could be used by those with low technical resources or skills.

**Conclusions:**

From our findings, we present a discussion of three key components of participants’ mental models, including the people, behaviors, and interactions they described that were important for IAs for consumer health information management and seeking. We then discuss the role of access, transparency, caregivers, and autonomy in design for addressing participants’ concerns about privacy and trust as well as its role in assisting others that may interact with an IA on the older adults’ behalf.

**International Registered Report Identifier (IRRID):**

RR2-10.1145/3240925.3240972

## Introduction

### Background

Advances in the field of artificial intelligence have led to growth in the number of consumer technologies that use intelligent assistants or intelligent agents (IAs) to help individuals with everyday tasks. The ubiquity of these technologies has led to a re-emerging interest in the use of IAs for aging and consumer health. IAs have the potential to provide older adults with new ways of managing their personal health and wellness decisions at home. Among the tasks that aging health care consumers often self-manage is the process of finding and making sense of health information to inform and provide self-awareness of their health and to support consumer health decisions.

Currently, many consumers rely on online health information to support health decisions and manage their health at home. A 2013 report of online health information seekers found that approximately 59% of respondents had searched online for health information for themselves or others [1]. In addition, access to online health information has been linked to improved health outcomes, especially among older adult populations [2]. Therefore, online health information is perceived as an essential resource to assist older adults with health care management and decisions [3]. Despite the potential benefits, many consumers still face challenges when searching for health information online [4-8]. Prior work has found that older adults face usability and accessibility challenges when searching for health information online and may find online health information overwhelming and have trouble understanding it [9-11].

### Older Adults and Online Health Information Search

The use of online health information by older adults to aid in health decisions has been largely beneficial. A large part of consumer decision making is the ability of an individual to use information they have gathered (prior knowledge) to inform their current decisions [12]. Older adults use online health information for a variety of reasons, including to support health decisions, search for information provided during doctor’s visits, and to manage disease [3,6,13]. A review of research regarding older adults’ online health information-seeking practices found that access to online health information was effective for improving several health outcomes (eg, adherence and overall quality of life) [2,14]. However, despite the many benefits, many older adults find it difficult to search for health information online.

Cline and Haynes [5] found that, in general, consumers face a myriad of challenges when searching for health information on the internet. Among these challenges are being presented with too much information, the use of technical language, and usability [4-8]. Because the information presented is often broad and difficult to navigate, users can also become confused or anxious [5,8]. Similarly, in the past, many online health websites were plagued with usability and accessibility challenges that made them difficult for older adults to navigate [9,10]. In addition to technical challenges, older adults have also been found to face other more general challenges related to understanding health information (ie, lower health literacy levels) and negative attitudes toward technology that can make it difficult for them to effectively make use of online health information resources [11]. Therefore, gaps in knowledge still exist on how to best support older adults’ consumer health information search practices and ways to help them find and understand the information they need to make informed consumer decisions about their health.

### Supporting Consumer Health Information Search

The emergence of new approaches for personalizing information and experiences has led to an increase in the number of intelligent interfaces that can assist with health tasks. The use of tailoring has been widely used in the area of health communication to reduce task complexity and simplify decision making among different groups of users [15]. However, researchers are beginning to explore how personalization, a form of digital tailoring, can be used to help improve online health search and communication tasks [16-23].

Several researchers have studied personalized approaches to support consumer search and understanding of health information [20,22-24]. For example, Fink and colleagues [24] explored the use of a guided search for assisting older adults with Web searches. They found that participants who used the guided search felt their search process would improve in the future. Several researchers have also looked at frameworks and models to support adaptive health interfaces [20,22,23] and further work on adaptive interfaces in health [16,21]. These interfaces automatically or semiautomatically change content or information based on knowledge of the user [25].

Shakshuki et al [20] proposed a multiagent learning technique for supporting adaptive health interfaces. Suggs and McIntyre [21] found that the availability of online tailored health communication for patients is increasing; however, it is not well-known what aspects of tailored communication contribute most to decision making. Similarly, Eslami and colleagues [16] found that although users were open to adaptation, it was important to identify the needs and preferences of users in context. Therefore, computer-based tailoring strategies are one way to support positive health outcomes [6,21,22], while also supporting the specific needs of users in the context of the health task [16].

Computer-based intelligent approaches, such as the use of IAs, represent an opportunity to personalize information, content, or processes to assist older adults with managing and finding relevant consumer health information at home. However, despite growing interest in IAs for aging and consumer health, and the importance of user perception on acceptance and adoption of emerging technologies, there are still significant gaps in literature regarding how older adults perceive IAs for consumer health, their perceptions of how IAs should behave and assist them, and how they would like to integrate IAs in their health care regimen at home. Gaining a better understanding of older adults’ beliefs and mental models of IAs for consumer health information management and search could lead to the design of tools that better align with their needs and better adoption and long-term use of these tools with potentially better health outcomes. The goal of this study is to explore older adults’ perceptions, challenges, and needs for assistance to identify design opportunities for intelligent interfaces to support them in this task.

## Methods

### Overview

To understand older adults’ perspectives regarding IAs for health information management and search, we conducted a design workshop with 18 older adult participants to identify their mental models. In this workshop, our goal was to better understand how older adults perceive an IA that would assist them with health tasks in their homes, including the physical form of the product (eg, how it looks), the function, and their beliefs and concerns about how it could be integrated and used within their home environment [26]. Two researchers assisted with the workshop. The workshop occurred over one day in July 2017 in a local senior center in Indianapolis, IN, and included several phases that involved different activities. We scheduled breaks between each phase to allow participants time to regroup and researchers time to prepare and transition to the next phase.

### Recruitment

We obtained institutional review board approval from Indiana University in Indianapolis, IN, before conducting the study. We recruited 18 participants from a local senior center. The only inclusion criteria were that participants be 60 years of age or older and have an interest in the purpose of the study. The senior center coordinator assisted with recruitment by sending our recruitment documents to their participant base, collecting names and contact information of interested participants, and helping to coordinate the workshop on-site. Written informed consent was collected on the day of the workshop. On arrival, each participant was provided with an informed consent document describing the purpose of the study, the study procedures, their right to leave the study at any point during the workshop, and contact information for the study principal investigator. In the session, participants were provided with time to read the informed consent or the option for the researcher to read the document to them. Participants were asked to sign the informed consent document if they were interested in proceeding. The workshop proceeded once all participants signed and returned their informed consent documents.

### Participants

Participants’ ages ranged from 61 to 93 years (mean 76, SD 8). Fifteen participants identified as female, and the remaining identified as male. Most participants (n=9) reported earning a high school diploma or equivalent (ie, GED), four participants earned an associate’s degree or equivalent, and five participants reported that they earned less than a high school education. All but one participant (n=17) was retired.

Most (n=8) participants self-rated their current health status as relatively healthy. Six participants rated their current health status as somewhere between healthy and not so healthy, and three participants rated their health status as not so healthy. One participant did not rate their current health status. Reasons participants listed for their rating of relatively healthy included participation in regular exercise and healthy eating, not having any ailments (ongoing chronic illnesses or health issues), minor ailments such as slightly elevated blood pressure due to stress and acid reflux, not taking much medicine for their age, and being a three-time cancer survivor (ie, being diagnosed with cancer three times and surviving each time). Reasons participants listed for a rating between healthy and not so healthy included trying to eat healthier, borderline diabetes, slightly elevated or chronically high blood pressure and cholesterol, arthritis, joint and back pain, and minor complaints. Participants that rated themselves as not so healthy noted their reasons as having a disease such as congestive heart failure, multiple chronic conditions (chronic obstructive pulmonary disease and diabetes), and having a myocardial infarction.

In addition to demographic questions, we asked participants questions about their technology use. Most participants (17/18, 94%) did not use technology regularly (defined as more than 5 days per week); however, 11 (61%) participants reported browsing the internet periodically (1-2 days per week) using a mobile phone, tablet, or computer. Two participants browsed the internet on a regular basis (more than 3 days per week). Most participants (14/18, 11%) used a basic cell phone (ie, without smartphone capabilities) regularly or more than 5 days per week. Two (11%) participants used a smartphone and two (11%) participants used a laptop regularly. Three (17%) participants used a desktop; three (17%) participants used a touch-based tablet such as an iPad regularly. Of those that tracked their health information, most used paper and pen, but two participants reported using a mobile app to track their health indicators and one participant each used a wearable fitness tracker (Fitbit), diabetic meter, and desktop software. Fewer participants used technology to manage or organize health information. Only two participants reported using a website or other technology to manage health information.

### Participatory Design Workshop

**Figure 1:**
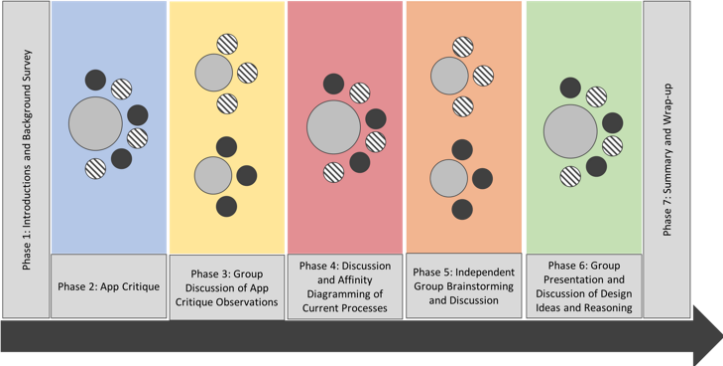
Diagram outlining the flow of the participatory design workshop.

The workshop was conducted in several phases with break periods interspersed between design activities to allow periods of rest for the participants and time for the researchers to organize data and prepare for subsequent phases (see Figure 1). On arrival, participants were greeted and provided with additional information about the study and a consent form. Once we obtained consent, we also asked participants to complete a demographic and computer use survey (phase 1).

Participants were then asked to critique WebMD on a mobile or Web-based interface (phase 2). We first introduced the app to the larger group, and participants were later asked to divide into groups to complete the critique activity. The purpose of the critique session was two-fold. First, the critique acted as an icebreaker for groups of participants to get to know one another. Second, we wanted to introduce the participants to the idea of considering the benefits and tradeoffs of a design to prepare for later design activities. Groups were given time to try the interface and discuss the benefits and challenges with their teammates. Participants were provided with a printed copy of a list of questions to consider, including their initial impressions of the interface, what they liked and disliked about the interface, and how they may or may not use it. One person in each group was also asked to take notes as their team reviewed the interface. At the end of the critique, each team presented their thoughts to the larger group of participants (phase 3).

Researchers then engaged participants in an affinity diagramming session (phase 4) to identify how they manage and search for health information, the challenges they face, and their use of technology to assist in the process. Affinity diagramming is a process in which individuals iteratively cluster opinions, experiences, or insights to keep design teams grounded in data [27]. One researcher facilitated the discussion while the second researcher took notes. Participants were asked questions about how they keep track of health information at home. Participants wrote responses on sticky notes and placed them in a common area. Afterward, one researcher led the group in a discussion of the responses as the other researcher continued to take notes.

After a short break, participants brainstormed ideas for an intelligent or “smart” interface that might assist them with finding and managing consumer health information (phase 5). To give some guidance on the definition of an IA, the facilitator provided a scenario that included a user interacting with a nontechnical form of assistance, such as asking a doctor to find health information. The facilitator explained that a smart interface might perform similar tasks. The facilitator also explained that they could think of a technology that could assist them with questions they had about their health. However, because we wanted to understand participants’ ideas of how an IA for health might look and work, the facilitator emphasized that their ideas could be any tool or product that they felt could assist them with achieving this goal.

For this part of the workshop, participants were divided into five groups of at least three team members and spent approximately 30 minutes brainstorming and discussing their ideas. Each group was again provided with a set of questions to help them think through the reasons behind their designs and to help us keep track of their reasoning. The questions focused on helping them think about what type of assistance they wanted, how they would use their technology, and the reasons for their decisions. One group member was asked to take notes to later report to the group.

Both facilitators walked around to listen in on discussions and to take notes. Toward the end of the design activity, each researcher visited briefly with each group to help them refine their ideas and prepare for presentation. After the design activity, each team presented their idea to the larger group for discussion along with their reasons for their decisions (phase 6). However, it is important to note that because the goal of the workshop was to understand participants’ beliefs and not to explore novel designs, we did not participate in the idea generation as to not bias our results. Finally, participants were asked for any additional feedback on the study, thanked, and provided with a US $20 gift card for their time (phase 7).

### Data Analysis

**Figure 2:**
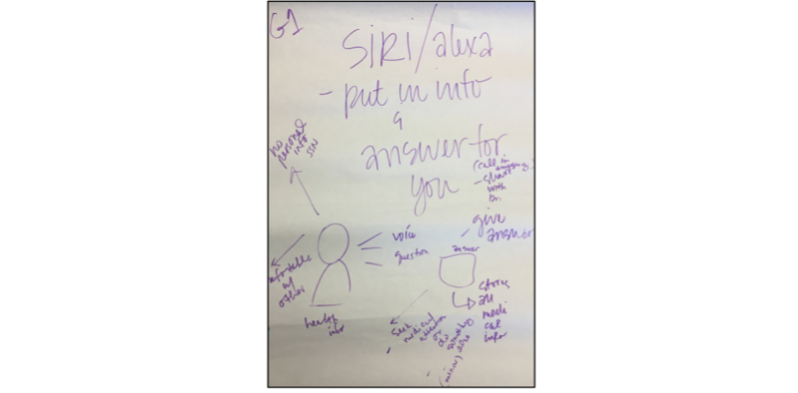
Brainstorming sketch from the design workshop for group 1.

Researchers took detailed handwritten notes of participant responses and their research observations. Immediately following the workshop, the two researchers who facilitated the workshop met to debrief and compare notes. The workshop sessions were audio-recorded and later transcribed to supplement researchers’ notes about participants’ responses to questions in group discussions and sketches of the participants’ design concepts (see Figure 2). All artifacts collected during the workshop, including large sticky notes of design concepts sketched during the workshop, images of the affinity diagramming results, and participants’ written descriptions of their ideas, were compiled to supplement the transcripts and notes. Data analysis involved open coding of data by three researchers to identify common themes in the data to create a list of codes [28]. Codes were iteratively refined and later applied to qualitative data. High-level themes were developed using axial coding. Data collected in the demographic survey were analyzed using descriptive statistics.

## Results

Themes emerging from the design workshop centered on older adults’ perceptions of their expectations of how IAs could be designed and used for consumer health information search and management, concerns they had regarding using IAs for consumer health search activities, and concerns about potential barriers that would limit their ability to integrate IAs in their home.

### Health Information Management Strategies and Challenges

The findings from the participatory design workshop’s affinity diagramming session revealed that 7 of 18 participants did not have strategies in place to manage their health information. However, during the discussions, most participants agreed that there was value in keeping track of health information themselves and, therefore, a combination of their interest in improving their health and past challenges with attempting to use technology to manage their health motivated their participation in the workshop. The advantages they discussed included scheduling, facilitating discussion with their doctor, staying informed, and being able to better monitor their health and identify a serious illness.

Of the 11 participants who did manage their health information, most used a paper-based filing system or calendar (n=5) or relied on their doctor to provide information about their health (n=4). Participants discussed several types of health information they tracked, including medication information, appointments, insurance information, and alternative treatments. Of those participants who searched for health information, most (n=6) used that information to consult with their doctor and also included at least one other person in their health care management routine. In addition to their doctor (n=6), participants discussed that they would also include immediate family members (eg, spouse or child) on issues related to their health (n=13).

Our participants saw value in keeping track of health information and being able to search for consumer health to support decisions and next steps. However, although most participant groups discussed that they had attempted at one point to find health information, not all participants currently actively managed their own health information or searched for health information at home.

### Participants’ Design Scenarios

Of the five groups of participants, four described ideas about IAs. One group (group 2) described their preference for talking with a health care provider or another caregiver in lieu of any other type of assistance. The design ideas presented by the groups were not completely novel as different aspects of the design have been addressed in other ways by technology. However, comparing the form, features, and functionality discussed and how participants described the assistance helped us to understand their perspectives of how they believed IAs for consumer health information management and search would look, behave, and be integrated into their lives. We did provide abstract guidance on what to design (IAs for assistance with health searches at home), and the participants also critiqued a website earlier in the workshop. However, similar to Davidson and Jensen [29], we found that the critique did not influence creativity of ideas and each group developed somewhat unique designs. We provide a brief description of each subsequently.

#### Intelligent Voice Assistant for Health

Participants in group 1 posed the idea of an intelligent voice assistant (eg, smart speaker) that they could ask health questions, and it would respond with appropriate answers. They discussed that their idea was inspired by commercials they had seen for Echo and Google Home, and they felt that this would be a good way to interact with health information. However, different from existing devices, the system would store their health history and provide answers that were specifically relevant to them. The system would provide options for them to easily share information with caregivers and could automatically differentiate between minor and severe medical situations to detect emergencies.

#### Talk With a Health Professional or Caregiver

Participants in group 2 expressed that they preferred to talk with a health care professional instead of interacting with an IA for health. They felt that talking to a health care provider would be faster for finding answers to health questions because the provider would already know their medical history. Participants had not experienced challenges with quickly communicating with their doctor in the past. They noted that their opinion might change if their providers were “very busy.”

#### Simple Interactions and Simple Information

Group 3 felt that the technology medium that communicated the information would not matter as long as it was easy to learn, use, and provided simple interactions. They described that the system might ask them questions (but not too many) about health conditions or symptoms and provide tailored search results. They also stressed that information communicated should above all be easy to understand and use simple language that is not overwhelming.

#### Q&A Health Website

Group 4 described a health website or “personal device” that could provide them with “simple” answers to the questions they asked. The inspiration for this design came from the participants’ experiences attempting to use the internet to find information, and the challenges they encountered using different websites. The website would not include any advertisements and could provide answers that were tailored to them. The assistant would also provide suggestions on other topics, such as how to manage their chronic illness or alternative medications to try.

#### Automated Phone System

Group 5 described an automated phone system for finding answers to health questions. The inspiration for this design came from participants’ beliefs that they felt not all older adults would have access to technology, such as an iPad, computer, or even the internet, but they felt that most would have a phone at home. They described that the automated system would store information about a user’s health history and emergency contacts, which they would enter during account setup. The user could then call the system to receive personalized answers to their questions, connect with local providers, or find information about symptoms or medications. The system could also compare symptoms with their health history to infer about and diagnose serious illness or emergencies.

### Types of Assistance Described

From the scenarios, five themes emerged related to the ways participants believed an IA might benefit or improve their day-to-day consumer health tasks at home. Because of past difficulties searching for health information at home, all the ideas proposed by the four groups were ideas for IAs that could make searching for health information easier (see Table 1).

Participants described how an IA might help them find relevant information faster by using knowledge of their health to provide tailored responses or narrow search results. For example, a member of group 3 expressed frustration with trying to find information relevant to their needs online:

[When searching for health information] Get to the point. I don’t want to have to [search through] 50 answers just get to the point. I mean I tried to get on sites [health websites] and everything...you know people say go here or something like that and you get there [to the website] and it says well you have to do this and you have to do this and this and this. Hey, you know, I just want to go there and get to the point.

Both groups 3 and 4 discussed the complexities of searching for and understanding health information. Therefore, their groups suggested features that could narrow choices and remove irrelevant information to support a straightforward search process. Groups 1, 4, and 5 proposed interfaces that could help with the search process by using knowledge of their medical history to provide personalized versus generic information.

Two groups also described in their scenarios instances in which an IA could help them make sense of health information by simplifying medical or health jargon and descriptions of health text. Groups 3 and 4 described a desire for features to help simplify the process of making sense of health information:

Explain things in plain language...currently it’s [health information] hard to understand

Group 3

Therefore, participants proposed IA features that not only helped them find relevant information faster, but that could also assist them with understanding the information once presented. Similarly, groups 1, 4, and 5 suggested that IAs could provide them with advice and recommendations about illnesses, symptoms, and medications. For example, when describing their design scenario, group 5 explained that their automated phone system would allow a user to “call in to ask a question about a symptom or illness and get an answer.” They described that the user would have a code that would allow them to store their information, and after entering the code they could “ask questions about some type of symptom they may have.”

**Table 1:** Types of assistance that groups mentioned when describing their intelligent assistant concept.

Type of assistance	Group(s)^a^
Finding relevant consumer health information	1, 3, 4, and 5
Making sense of consumer health information	3 and 4
Providing advice/recommendations	1, 4, and 5
Facilitating collaborative decisions	1, 3, 4, and 5
Diagnosing serious or emergency illnesses	1 and 5

^a^Group 1: intelligent voice assistant for health; group 3: simple interactions and simple information; group 4: Q&A health website; group 5: automated phone system.

Although each group brainstormed their design scenarios independently of other groups and the researchers, the mention of “simple search,” “simple language,” and “simple direct answers” were pervasive as each group shared their design scenarios with the larger group. The participants’ desire for simplicity was mainly due to their experiences and perception of the complexity of the online search process for health information. Therefore, most participant ideas centered on how IAs could support the search process by removing some of that burden from the user. However, groups were not fully trusting of IAs for certain search tasks as evidenced by their dialog on the importance of including functionality that allowed the system to facilitate collaborative decisions about their health with a doctor or family member. All groups mentioned features that allowed them to collaborate with doctors or family members involved in their caregiving and health care decision making:

We were thinking [initially] a personal device [the interface idea], but maybe it could share with the doctor or family that you would want to include in decision-making processes

Group 4

The spokesperson for the group indicated that they originally felt that the interface should include some sort of option for storing information locally, so that the user could limit access to their medical information and preferences; however, they decided that it would be useful to share information with others that could provide input to the users’ decisions. Groups 1, 3, and 5 agreed that there would be cases in which they would prefer or feel more comfortable talking with a health care professional. They indicated that their interface idea included features that would allow them to share information and include either a health care provider or family member in their decisions if they desired.

Finally, two groups discussed scenarios in which their assistant would help with the diagnosis of serious illness (eg, congestive heart failure) by learning about their health and reasoning from their queries:

[I would include] all my medical information, my medical history, like if I have congestive heart failure and if I am having pains or something, I could ask it something and it could tell me if I need to seek medical help or maybe it could get me something that I could use to ease it myself.

Group 1

### Proposed Technology Medium and Ways of Interacting

Each of the four groups that presented ideas for IAs introduced different mediums, including a voice assistant, website, and an automated telephone system; one group was apathetic about the medium but stressed that it should be simple, easy to learn, and easy to use. Most of the discussion about form centered around the need for IAs to be integrated into technology that is familiar or that provides for natural interactions that are easy to learn. Some participants also discussed the need for the technology to be easily accessible to those with and without technology resources at home. However, we also found that the groups described similar qualities when discussing their assistant and how it would work. The purpose of the workshop was to understand older adults’ mental models of IAs for health; therefore, each group (except for group 2), proposed some level of intelligent interaction. Apart from intelligence, groups seemed to describe mediums with which they were either already familiar (website, telephone) or that could be easily learned through natural interaction (voice assistant, simple medium):

They have these books for everything. So, we put down “Health Info for Dummies”...It could be a website or whatever...Put it [the information] in simple language so that people will know what it is. Also, not 50 pages of blah blah blah...just simple, simple language, easy to use.

Group 3

Participants also described fluid and unconstrained interactions with their assistant noting at times that they felt the assistant should reason about their symptoms, provide recommendations or information, and seamlessly move from one health topic to the next:

A machine like Alexa, [you] put in all the medical information that pertains to seniors like arthritis, headaches, broken hips, and all that stuff...we ask it a question and it answers it...You know, [you can say] I have a headache, I have this, I have that. Give them the symptoms just like you do on a tablet, and it will come up with the answer.

Group 1

At the same time, groups also described the need for the assistant to be transparent about its limitations for providing safe advice and instead be able to switch from the role of assisting with care to facilitating care:

If it’s not serious [the situation], the system could instead provide them with a list of doctors names and numbers that they could contact in their area for help

Group 5

Therefore, although participants described different mediums, they all seemed to value familiar and natural mediums that were easy to learn and use. In addition, groups seemed to value interactions that were transparent but also fluid and unconstrained.

### Concerns for Home Integration

Participants discussed two major concerns about integrating IAs for health in their homes. First, groups expressed concerns about privacy and described ways their IA might secure user information:

If there was some way of being able to store your own information on your own device. You know like...if you have access to the internet then they have access to your answers too, so I don’t like that, but if there were some way of cutting that out where you can access the Web without having to give it back all your information it would be nice to have your own personal thing. I don’t know how that would work.

Participant, group 4

All four groups raised similar concerns about privacy. Another group suggested using a code to limit access to personal health data using their automated phone system: “I’m just speaking for the people that don’t have access to the internet, so the phone would be ideal for them, and they could put in a code or something if they didn’t want people to get to that information” (Participant, group 5).

Participants raised a different set of concerns about internet access and availability at home and potentially being limited to certain mediums. Group 1, when brainstorming ideas for a solution, eventually settled on a voice assistant but considered their past challenges of finding a stable internet connection. They considered that other older adults may not have internet access at home and that they might have to negotiate for technology resources to be able to adopt the IA for health. The following conversation occurred between two participants in group 1:

PA: I don’t think a lot of seniors have Wi-Fi.

PB: They don’t because I come here [to the center] and then I can use it [the internet] here but I don’t have it at home. But, my neighbor across the street...

PA: Yeah, sometimes you can pick up on.

PB: So, I went over there [the neighbor’s house]...

PA: Like the folks next door?

PB: You have to get their Wi-Fi [password]...

PA: Right.

PB: and I did go over and ask them could I, you know, did she have Wi-Fi and she said yes, now since she told me, I can use this [the Wi-Fi] at home.

Another participant expressed that she also wished that there was not such a reliance on internet access when using different apps:

I just wish it [health apps] worked without the internet because I let mine [internet] go because my computer messed up and I was like oh well, I’m just not going to get another one [computer]. I [now] ask the kids something and they always Google it or do whatever they do to try and find out [for me]. They will try and find out from different people the symptoms or what they do to cure it, but that’s not actually what it is.

Group 5

Participants’ choice of medium and concerns for adoption also seemed to take into account whether or not they felt the system could be seamlessly adopted into their existing home environment without consequence.

## Discussion

We provide a summary of our findings of participants’ beliefs and mental models regarding the use of IAs for health information search and management at home.

### Modeling Interactions Between Older Adults and Intelligent Assistants for Health Information Management

**Figure 3:**
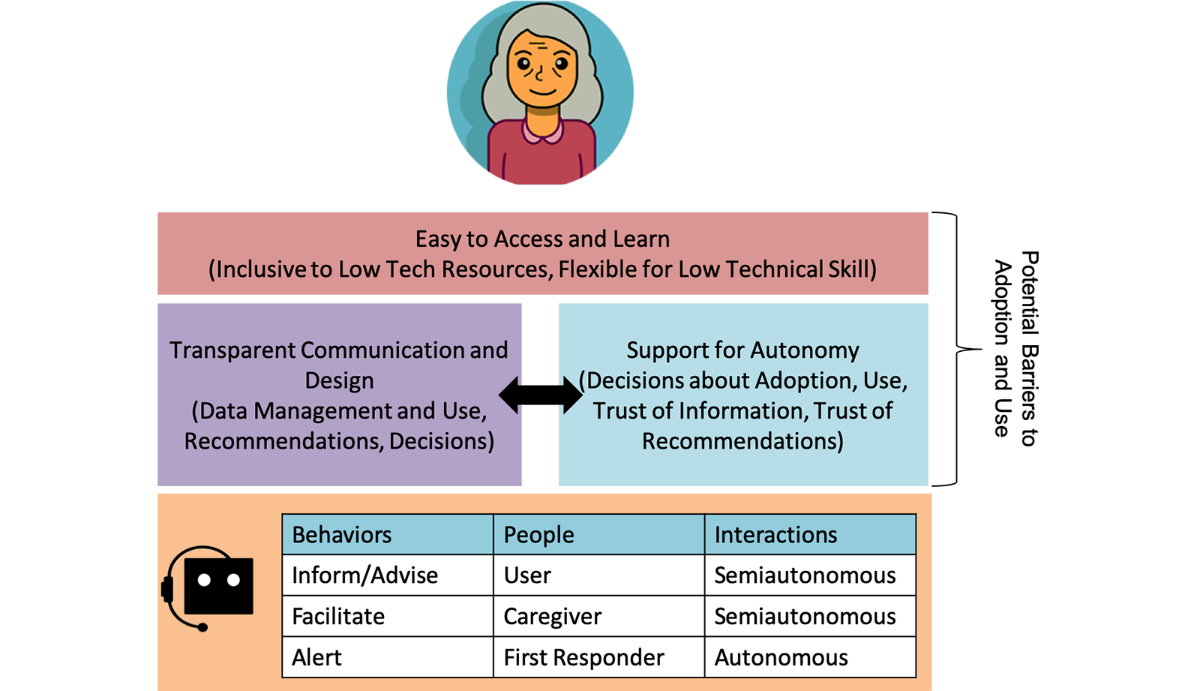
Model of participants' desired interactions with intelligent assistants for health information management and search.

Overall, our participants desired an IA that could reduce the time and effort it takes for them or others involved in their health care regimen (ie, caregivers, doctors) to find, manage, and share consumer health information relevant to their needs. Several groups discussed ideas related to the use of tailoring or personalization to improve search, navigation, or response. In general, the participants who suggested a technological solution wanted IAs that addressed some of the challenges they encountered with health information management and search that they felt were not currently being addressed or could be better addressed with an intelligent interface. From our findings, we contribute a model describing the categories of behaviors, people, and types of interactions participants expected from an IA for health as well as how participants expect these interactions to take place (see Figure 3).

In the model, the first layer relates to ease of access and learning. Before considering other factors, the ability to access the features provided by an IA for health, whether it be access to the IA in a low-tech resource environment or access by someone with low technical skill, can influence whether the IA is adopted. The discussions about the look and feel (form) of the IA centered around mediums that participants were already familiar with or natural (ie, perceived easy to learn) to emphasize the notion that interacting with an IA should not be cumbersome and should limit or eliminate their current challenges with health information management and search tools they had tried opposed to making the process more difficult.

The second layer focuses on transparency and autonomy, which not only plays a role in the potential adoption of an IA for health but also for long-term continued use. If older adults need to understand how the IA works and the need for governing the tasks supported by IAs are not met, it may influence initial adoption or use over time. We categorize access, transparency, and autonomy as potential barriers to adoption and long-term use because our findings suggest that these factors are often considered apart from the type of support provided. An IA may provide adequate support for a health information management or search task, but if it is not easily accessed, learned, or does not provide the proper levels of transparency and autonomy, our findings suggest it may not be adopted or may ultimately be abandoned. For example, a user may want help understanding what data are shared about themselves and, ultimately, if a certain IA feature is something they would like to adopt.

The third level represents communication and interaction between the user and the IA or other individuals involved in their care. Our findings suggest that the relationships between the three categories of behaviors, people, and interactions were somewhat fluid and reciprocal in that each category in some way related to and was dependent on the others. The behaviors described as tasks for IAs were sending alerts, facilitating interactions with others involved in their care, and informing or advising personal health decisions. The people participants discussed apart from themselves as being potential users that might interact with an IA on their behalf were informal caregivers (eg, child, spouse), formal caregivers (eg, doctor, nurse), and first responders (ie, emergency medical providers). Interactions described were either autonomous (ie, completed by the IA without their involvement) or semiautonomous (ie, completed by the IA with their involvement). For example, the ideas presented regarding IAs for alerts mainly focused on first responders; in this situation, participants desired more autonomous interactions that could initiate assistance if they were unable to do so themselves. When describing IAs that helped with the facilitation of health tasks, the discussion centered on formal and informal caregivers and the exchange of information for assisting them with awareness and decision making. In these instances, participants described more semiautonomous interactions in which they had control over what information was shared and when.

### Relationship to Prior Work

Some of the open issues that emerged from our findings are known or have been addressed in other fields of study. For example, participants discussed their desire for tailoring and personalization. In the field of health care, the idea (and practice) of tailoring information has been used for some time to provide personalized content to health care consumers [15]. In computer science and human-computer interaction, intelligent interfaces that gather user characteristics automatically or manually have been widely leveraged to adapt and provide users with personalized experiences [20,22-24]. Therefore, it is well-known that using IAs to personalize or adapt information and content can simplify the process for users. Therefore, our work builds on this prior work citing participants’ desires for personalized features in the design of consumer health information management and search tools. However, the success of any intelligent interface design and implementation project often largely depends on understanding users’ goals and needs for that specific task [19,25]. Our work contributes insight and understanding regarding how older adults perceive IAs might be useful to assist them with consumer health information management and search tasks at home. These insights can begin to help designers and researchers understand where implementation of IAs might likely yield adoption in this context. However, more research is needed to completely understand how to address these needs in a way that provides the transparency and autonomy desired, but that also considers other factors such as safety.

### Design Considerations: What Do These Findings Mean for the Design of Intelligent Health Information Management Tools for Older Adults?

We preface our discussion of design implications by revisiting the focus of this research, which was to explore older adults’ mental models and beliefs regarding IAs for consumer health and, specifically, IAs in the context of health information management and search. Therefore, we did not discuss IA for use in hospital or formal medical settings apart from supporting interaction with formal caregivers. We also acknowledge that some of the ideas presented by the groups may not seem novel; however, our goal for the participatory design session was not to develop novel tools or critique the participants’ designs, but to learn through the design sessions about participants challenges, concerns, and to identify considerations for future design.

### Addressing Current Challenges and Motivating Use Through Autonomy

Human-computer researchers have emphasized the importance of understanding users’ goals and expectations for automation when designing intelligent interfaces [19,25]. Our findings highlight several areas in which participants felt support from an intelligent agent might be useful to them. Many of the areas discussed stemmed from prior and current challenges they experienced managing and searching for health information in a consumer setting. Although some of these challenges have been addressed in prior work, it may be useful for designers and researchers in the future to better understand why participants have not considered adopting these solutions. More research on current approaches to addressing the highlighted areas and the benefits and tradeoffs can help researchers better understand the role of automation and whether it meets users’ needs and expectations. In addition, understanding how the individual expects to govern the task can help further identify areas in which IAs might be most appropriate and also how to design these assistants in a way that supports users’ goals for autonomy.

### Leveraging Relationships With Health Care Providers and Caregivers

Most participants described the importance of being able to engage with a doctor or another health care professional if needed. Groups that proposed these solutions were aware that there might be cases in which they would prefer or feel more comfortable talking with a health care professional. In addition, because health care providers and other caregivers often participate in collaborative decision making [3], an IA that could leverage these relationships and improve these collaborations may be beneficial, particularly to older adult users or other users that rely on these relationships to manage their health.

In parallel, it can also be useful to explore the role of intelligent interfaces for facilitating the exchange of information between formal and informal caregivers. Although we do not anticipate it to be desirable for an interface to fully replace the role of caregivers in consumer health decisions, there are opportunities to explore how these systems can better support the relationship between stakeholders, the exchange of information, and the steps leading to the decision to better empower the consumer. Further, similar to exploring how these interfaces might impact health care providers and caregivers, it would be important to also consider the effect they may or may not have on relationships and the decision-making process.

### Providing Intelligent Assistance Through Familiar and Accessible Mediums

The adoption of health technologies and the use of the internet for health information is growing among older adults [1]. However, there is still significant concern about older adults’ access to technology, in particular when related to access and internet skill [30]. Our participants expressed similar concerns about whether tools that include IAs would be accessible to them due to limited technical resources at home or limited technical skills. Therefore, a common theme from the designs from our older adult participants was that intelligent health tools must provide flexible access, but also be accessible to individuals that may have limited technical skill.

With the emergence of intelligent voice assistants, such as Siri or Alexa, the move to more natural interaction is already underway. However, more work will be needed to understand if these types of assistants can be useful in the context of health information management and search. Another key consideration will be how we can design IAs for health that support older adults without requiring a new device or technology. Although some participants expressed they would consider adopting a new medium, others discussed concerns about cost and infrastructural barriers that might limit their access and use of IA for health in a consumer setting. Participants’ perceptions were that IAs are data-intensive and rely heavily on a stable internet connection to facilitate interactions. Therefore, the need for internet would be a barrier for adoption for some. Exploring inclusive designs that address the varying needs of older adults may lead to more widespread access to and adoption of IAs that assist with consumer health information management and search practices.

### The Role of Transparent Design for Supporting Users

**Figure 4:**
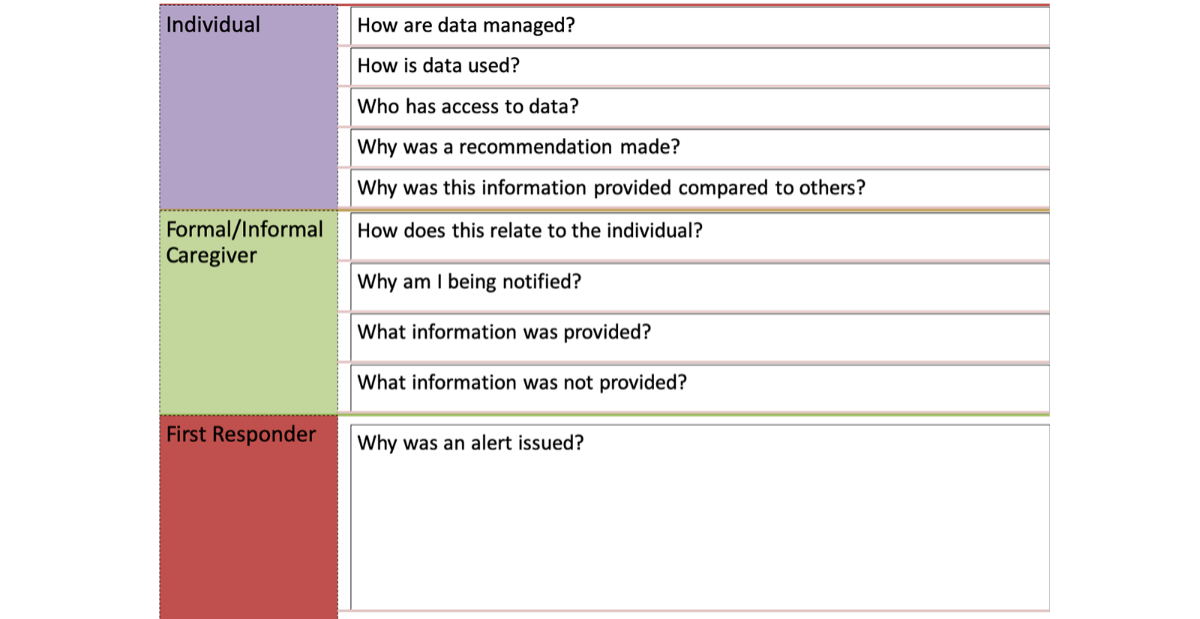
Example of questions to improve the transparency of system actions for older adult users and those involved in their care.

Emphasis on designing intelligent systems that are transparent and easy to understand has increased in recent years. One key theme that emerged directly and indirectly in the workshop was the importance of being able to understand system actions. All our participants valued privacy and trust, and those that proposed technical solutions emphasized the need for privacy and trust in their discussions. Explainable interfaces are one approach re-emerging to improve transparency [31]. In addition, processes for creating transparent designs have been recently proposed [32]. However, there is still a lot we do not know about how to design interfaces that support this transparency. Figure 4 summarizes the different types of transparency mentioned by participants in the discussions as a first step to understanding what participants want to be explained [32]. In addition, we include potential questions of interests to other stakeholders who may interact with an IA on behalf or in collaboration with the participant (see Figure 4).

Participants raised concerns about how an IA would secure and manage their data. Participants also expressed concerns about being able to trust system recommendations and the situations in which a system recommendation may need further confirmation from a health care provider. However, because the participants envision that it would be responsible and useful for caregivers and first responders to also interact with an IA on their behalf, we expect that there may also be ways to help them better interpret the information and recommendations provided to them. If the user wants to discuss something with their doctor, it may be helpful to provide the doctor with information about why the IA provided certain information or did not provide other information to assist the doctor in their discussion. Overall, with a focus on health, it will be important for IA designers to explore methods for helping users and others involved to understand how their data are used and managed as well as how recommendations are made. In addition, given the potentially diverse abilities of older adults, there may also be a need to explore how to approach the design of these interfaces in a way that supports their diverse and changing abilities.

### Limitations and Future Work

Our study represents an exploratory step in understanding older adults’ perceptions of intelligent interfaces that assist them with consumer health information tasks at home. The needs and desires for health information search support at home that are described in this paper are limited to the participants that were involved in the design workshop and their unique experiences and challenges. Also, because our study only focused on consumer health information search and management tasks, the findings may or may not apply to other contexts. It is possible that there are wider ranges of needs or desires for support that were not identified. Additionally, many of the older adults in our study were limited technology users and expressed challenges with searching for health information in the past. Therefore, it is possible that older adults who use technology more regularly may have different ideas about how technology might assist them. In the future, we will continue to explore the design of personalized tools to support older adults’ health decisions. One of our future goals is to include caregivers in discussions about IAs for health information search. Although this study focused only on older adults’ beliefs, we did find that most of our participants (n=13) currently included caregivers in their health information search and management process at home. In the future, it will be useful to include caregivers’ perspectives as well. Further, we will build on the findings of this study to design tools and evaluate them with older adults. We are exploring one of the ideas (voice assistants) discussed by participants as an option for delivering health information to better understand the contextual factors that exist around interacting with health information using voice.

### Conclusion

In this paper, we present findings from a participatory design workshop in which older adults brainstormed and conceptualized ideas for technology to assist them with consumer health information management and search at home. Five groups of older adults (N=18) brainstormed and described scenarios of ways an intelligent interface solution could or could not assist them in finding information and searching and managing health information in a nonclinical setting (ie, at home outside the doctor’s office). Four of the five groups presented solutions involving technology, whereas one group expressed their desire to forgo any type of software intervention and talk directly with their health care provider.

Our findings suggest that older adults have clear beliefs about how IAs might assist them with health information management and search. Although participants saw the benefit of IAs for health, older adults had concerns related to autonomy and transparency in design. Our research identifies a set of key factors that older adults perceive as important in the design of an IA for health. Because the perception of benefit (ie, perceived benefit) is a key factor when older adults make decisions to adopt a technology [33], the initial step of understanding beliefs regarding IAs for health is important to designing technologies that are likely viewed to provide benefit to older adults. Therefore, this work contributes (1) a better understanding of older adults’ mental models toward IA for health and (2) a set of initial considerations for designing IAs that assist older adults with health information search and management. Although our focus is older adults and some aspects (eg, the role of caregivers) may apply differently in other contexts, we anticipate that our findings can help inform the design of IAs that support others in managing and searching for health information at home. In addition, the discussion of participants’ expectations, experiences, and interface support needs can help designers, researchers, and developers of consumer health search interfaces brainstorm and identify solutions that address these challenges.

## Abbreviations

IA: intelligent assistant or intelligent agent

## References

[1] (2013). Pew Research Center.

[2] Bolle Sifra, van Weert JC, Daams J, Loos E, de Haes HC, Smets E (2015). Online health information tool effectiveness for older patients: a systematic review of the literature. J Health Commun.

[3] Xie B (2009). Older adults' health information wants in the internet age: implications for patient-provider relationships. J Health Commun.

[4] Adams S (2010). Revisiting the online health information reliability debate in the wake of. Int J Med Inform.

[5] Cline R, Haynes K (2001). Consumer health information seeking on the Internet: the state of the art. Health Educ Res.

[6] Hall A, Bernhardt J, Dodd V (2015). Older adults' use of online and offline sources of health information and constructs of reliance and self-efficacy for medical decision making. J Health Commun.

[7] Joe J, Demiris G (2013). Older adults and mobile phones for health: a review. J Biomed Inform.

[8] Lee K, Hoti K, Hughes JD, Emmerton LM (2015). Consumer use of "Dr Google": a survey on health information-seeking behaviors and navigational needs. J Med Internet Res.

[9] Nahm E, Preece J, Resnick B, Mills ME (2004). Usability of health web sites for older adults. CIN-Comput Inform Nu.

[10] Becker SA (2004). A study of web usability for older adults seeking online health resources. ACM Trans Comput-Hum Interact.

[11] Berkowsky R, Czaja S, Pak R, Collins Mclaughlin A (2018). Challenges associated with online health information seeking among older adults. Aging, Technology And Health.

[12] Bettman J, Johnson E, Payne J, Robertson TS, Kassarjian HH (1991). Consumer decision making. Handbook of Consumer Behavior.

[13] Flynn K, Smith M, Freese J (2006). When do older adults turn to the internet for health information? Findings from the Wisconsin Longitudinal Study. J Gen Intern Med.

[14] Shim H, Ailshire J, Zelinski E, Crimmins E (2018). The Health and Retirement Study: analysis of associations between use of the internet for health information and use of health services at multiple time points. J Med Internet Res.

[15] Kreuter M (2019). Tailoring Health Messages: Customizing Communication with Computer Technology.

[16] Eslami M, Firoozabadi M, Homayounvala E (2017). User preferences for adaptive user interfaces in health information systems. Univ Access Inf Soc.

[17] Jimison H, Pavel M, Pavel J (2003). Adaptive interfaces for home health.

[18] Lustria M, Cortese J, Noar S, Glueckauf R (2009). Computer-tailored health interventions delivered over the Web: review and analysis of key components. Patient Educ Couns.

[19] Miller CA, Funk H, Goldman R, Meisner J, Wu P (2005). Implications of adaptive vs. adaptable UIs on decision making: Why “automated adaptiveness” is not always the right answer.

[20] Shakshuki E, Reid M, Sheltami T (2015). An adaptive user interface in healthcare. Procedia Comput Sci.

[21] Suggs L, McIntyre C (2009). Are we there yet? An examination of online tailored health communication. Health Educ Behav.

[22] Vasilyeva E, Pechenizkiy M, Puuronen S (2005). Towards the framework of adaptive user interfaces for eHealth.

[23] Vogt J, Meier A (2010). An adaptive user interface framework for eHealth services based on UIML. https://aisel.aisnet.org/bled2010/13.

[24] Fink A, Beck J (2015). Developing and evaluating a website to guide older adults in their health information searches: a mixed-methods approach. J Appl Gerontol.

[25] Jacko JA, Sears A, Byrne M, Card S, Cockton G (2019). Adaptive interfaces and agents. The Human-Computer Interaction Handbook: Fundamentals, Evolving Technologies And Emerging Applications, Third Edition (Human Factors And Ergonomics).

[26] Martin-Hammond A, Vemireddy S, Rao K (2018). Engaging older adults in the participatory design of intelligent health search tools. Proceedings of the 12th EAI International Conference on Pervasive Computing Technologies for Healthcare.

[27] Hanington B, Martin B (2012). Universal Methods Of Design: 100 Ways To Research Complex Problems, Develop Innovative Ideas, And Design Effective Solutions.

[28] Corbin J, Strauss A (2012). Basics Of Qualitative Research: Techniques And Procedures For Developing Grounded Theory.

[29] Davidson J, Jensen C (2013). Participatory design with older adults: an analysis of creativity in the design of mobile healthcare applications. Proceedings of the 9th ACM Conference on Creativity & Cognition.

[30] Hargittai E, Piper AM, Morris MR (2018). From internet access to internet skills: digital inequality among older adults. Univ Access Inf Soc.

[31] Pu P, Chen L (2006). Trust building with explanation interfaces.

[32] Eiband M, Schneider H, Bilandzic M, Fazekas-Con J, Haug M, Hussmann H (2018). Bringing transparency design into practice.

[33] Melenhorst A, Rogers WA, Bouwhuis DG (2006). Older adults' motivated choice for technological innovation: evidence for benefit-driven selectivity. Psychol Aging.

